# Hypothesis Testing of Population Percentiles via the Wald Test with Bootstrap Variance Estimates

**DOI:** 10.4236/ojs.2016.61003

**Published:** 2016-02-14

**Authors:** William D. Johnson, Jacob E. Romer

**Affiliations:** Department of Biostatistics, Pennington Biomedical Research Center, Louisiana State University, Baton Rouge, LA, USA

**Keywords:** Bootstrap, Hypothesis Testing, Nonparametric Methods, Percentile Profiles, Wald Test

## Abstract

Testing the equality of percentiles (quantiles) between populations is an effective method for robust, nonparametric comparison, especially when the distributions are asymmetric or irregularly shaped. Unlike global nonparametric tests for homogeneity such as the Kolmogorv-Smirnov test, testing the equality of a set of percentiles (*i.e*., a *percentile profile*) yields an estimate of the location and extent of the differences between the populations along the entire domain. The Wald test using bootstrap estimates of variance of the order statistics provides a unified method for hypothesis testing of functions of the population percentiles. Simulation studies are conducted to show performance of the method under various scenarios and to give suggestions on its use. Several examples are given to illustrate some useful applications to real data.

## 1. Introduction

When comparing the distributions of a random variable observed under different conditions, there often is interest in testing equality of the parameters that circumscribe the respective central tendency of the condition-dependent observations. Thus, a standard t-test is often used when the data are (approximately) normally distributed, although under certain conditions, other parametric models may be used. Parametric tests may be adequate under some conditions, but there are many situations where the underlying distributions have irregular forms and parametric approaches to testing for distribution equality are inappropriate. When the underlying parametric distribution is unavailable or ignored, nonparametric methods such as the Mann-Whitney U test or Kolmogorov-Smirnov (KS) test are often employed to test the null hypothesis that the sample data come from random variables that have identical distributions, regardless of the imposed circumstance. Tests for equality of location may be too narrow in that they test a single parameter and ignore other aspects that often distinguish the distributions. On the other hand, global tests of equality such as the KS test are too broad and do not specifically portray where the distributions have significant discrepancies. Use of percentiles (quantiles) may be uniquely informative for comparing skewed distributions that do not have similar shapes.

For instance, suppose a treatment causes a substantial shift in only one segment of the population, such as those with biomarker levels in the range of the 90th percentile or above, and is virtually ineffective for those with levels below the 90th percentile. A location test may be less likely to detect this difference. Testing the equality of several, pre-specified percentiles (a *percentile profile*) along the entire range of the distribution will be insightful. In this paper, we illustrate the use of the Wald test employing variance estimates obtained via bootstrap samples to test for equality of population percentiles. In this way, it is possible to test the statistical significance of differences along the entire range of the distribution. Similarly, we can construct confidence intervals for the population-level differences for each percentile between the respective distributions. Statistical inferences based on percentile estimation are also advantageous in that the methods are robust to outliers and can be used when the data have complexly-shaped distributions.

**Figure 1:**
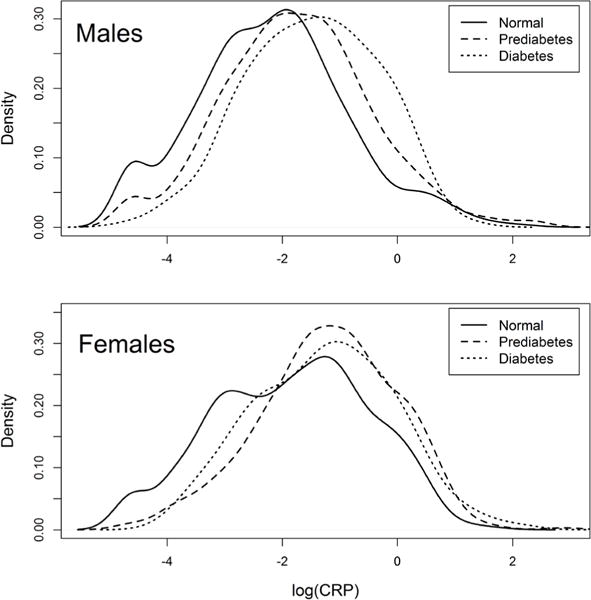
Kernel density estimates of log(CRP) of normal, prediabetes, and diabetes groups for males and females.

A motivating example (which we explain in detail in Section 5) comes from the National Health and Nutrition Examination Surveys (NHANES), a program of studies to monitor the health of adults and children in the United States (http://www.cdc.gov/nchs/nhanes.htm). One variable of interest is C-reactive protein (CRP) in adults—mainly, whether the distribution of CRP differs with diabetes diagnosis. Plotting the empirical density of log(CRP) for adults who are normal (do not have prediabetes or diabetes), adults who have prediabetes, and adults who have diabetes clearly reveals differences in distribution and hence percentiles (Figure 1). A median test or Mann-Whitney U test will detect some change in the median location between diabetes diagnosis groups, but this may be insufficiently informative due to the unusual shape of the data. To truly understand the differences, we require comparisons spanning the entire domain of a relevant biomarker for diabetes.

Johnson *et al*. [1] developed an extension of the median test to simultaneously test the homogeneity of any number of percentiles between groups. In this procedure, the samples from all groups are combined and common percentile estimates are found (as in the median test). These percentile estimates are used as cutoff points to sort the data from the respective comparative groups into columns of a contingency table where each row contains categorized data for a specific group. The authors then applied Pearson’s chi-square test to assess the homogeneity of the groups with respect their percentile profiles. The procedure proposed in [1] provides a heuristic and easy to use approach for performing overall joint tests of equality of a set of percentiles among multiple distributions, but it is limited by awkward methods for testing sub-hypotheses and constructing confidence intervals on differences and other functions of the percentiles of interest. Formulating the problem as a Wald statistic and using bootstrap estimates of the covariance matrix of the population percentiles provides unified methods for statistical testing and confidence interval construction.

There has been extensive work in quantile estimation and estimation of variance of quantiles with use of bootstrap resampling [2]–[7]. Bootstrapping is an invaluable tool to obtain estimates of variance of statistics, such as sample quantiles/order statistics, that are difficult to estimate without model parametrization. Wilcox [8] outlined a similar procedure for comparing quantiles of two independent samples by utilizing bootstrap estimates of variance. Hutson and Ernst [9] derived closed-form solutions for the exact bootstrap mean and variance of *L*-estimators, which could be applied to the present problem. In this paper, we formulate the statement of a general linear hypothesis in terms of linear functions of a population vector of percentile profiles. We also formulate a Wald statistic for testing this general hypothesis. This test statistic has a distribution that approaches that of a chi-square when the original sample sizes are sufficiently large. One advantage of the Wald test is it provides the ability to test multiple parameters jointly and construct contrasts on the functions of the population percentiles in terms of vectors and matrices. It can also be used with estimates from any percentile estimator and bootstrapping plan (*n* out of *n*, *m* out of *n*, etc.). This flexibility and ease of use make the Wald test a natural method for testing equality of percentiles and constructing confidence intervals on functions of percentiles. Its practicality is evident in large-sample applications, as illustrated by examples.

The paper is organized as follows: Section 2 explains the details of the procedure as well as a brief numerical tutorial. Section 3 provides some details of bootstrap sampling methods with respect to sample percentile covariance estimation. Results of selected simulation studies are presented in Section 4 followed by an illustrative example in Section 5 in which we explore the motivating example (differences in CRP distribution among diabetes groups) by testing several hypotheses. Section 6 is left for concluding remarks and discussion of further research.

## 2. Methods

### 2.1. Inference for a Single Percentile

Suppose we wish to construct a confidence interval for a population percentile which is defined as the value of the quantile function *Q*(·) evaluated at some *u*. Thus, *Q*(*u*) is a quantile of interest and *q*(*u*) is its sample estimator. Note that *Q*(*u*) can be estimated a number of ways (we henceforth refer to *q*(*u*) and *Q*(*u*) as simply *q* and *Q*, respectively). We define $q=X_{\left(\lfloor nu\rfloor +1\right)}$, where *X* is the sample, *n* is the number of observations, ⌊·⌋ denotes the floor function, and *X*(*r*) denotes the *r*th sample order statistic. By this definition, estimation of the population percentile and estimation of the percentile variance are equivalent to estimating the expected value and variance of the sample order statistics. With this in mind, it is possible to use linear combinations of sample order statistics as percentile estimators, though here we only focus on using single order statistics. We can obtain an estimate of the variance of *Q* by generating *B* bootstrap samples and obtaining *q* for each bootstrap sample, which is simply the (⌊*nu*⌋+1)th order statistic in the bootstrap sample. Without loss of generality, let ${\tilde{q}}_{h}$ denote the estimate of *Q* for the *h*th bootstrap sample. The usual variance estimator *v* is

$$v={\left(B-1\right)}^{-1}\sum_{h=1}^{B}{\left({\tilde{q}}_{h}-\bar{\tilde{q}}\right)}^{2}$$

where *B* is the number of bootstrap samples and $\bar{\tilde{q}}$ is the mean of the bootstrap estimates of *Q*. The (1–*α*)% confidence interval for *Q* can be calculated as *q*±*z*_1–α/2_*v*^1/2^. Note that if *q* is defined as a linear combination of the sample order statistics the appropriate covariance must be used.

If a second sample is available from an independent population and we wish to construct a confidence interval on *Q*_1_ – *Q*_2_, the difference of *Q* between the populations, we follow a similar procedure. First, obtain the sample estimates of *Q* for the first and second population: *q*_1_ and *q*_2_, respectively. Similarly, generate *B* bootstrap samples from each of the respective samples and calculate the variance of the ${\tilde{q}}_{h}’s$ (*h* = 1,⋯, *B*) for each population (as above). Let *v*_1_ and *v*_2_ denote the bootstrap variance estimate of the first and second populations, respectively. The (1–*α*)% confidence interval for *Q*_1_ − *Q*_2_ can be calculated as (*q*_1_−*q*_2_)±*z*_1–_*_α_*_/2_ (*v*_1_+*v*_2_)^1/2^ (assuming the populations are independent and cov(*Q*_1_,*Q*_2_) = 0).

### 2.2. Comparing *p* Percentiles from *K* Populations

Suppose we are given a random sample from each of *K* independent groups and we wish to test the equality of the *K* groups with respect to their percentile profiles, where each profile consists of a set of *p* percentiles. Denote the percentiles to be tested as ***Q****_i_* = (*Q_i_*_1_,⋯,*Q_ip_*) where *i* = 1,⋯, *K*. The method proceeds as follows:

**Step 1.** Estimate the percentile profile of interest for each of *K* populations, where the estimate is denoted as ***q****_i_* = (*q_i_*_1_,⋯, *q_ip_*). Recall that the *j*th percentile estimate may be a single sample order statistic corresponding to ⌊*nu_j_*⌋ + 1 (*j* = 1,⋯,*p*) or a combination of multiple order statistics.
**Step 2.** Arrange the percentile estimates in a *Kp* column vector, ***q***, where ***q***′ = [***q***_1_|⋯|***q****_K_* ].
**Step 3.** Generate *B* bootstrap samples from each of the original samples and estimate ***Q***, the set of percentiles of interest, on each bootstrap sample. Let ${\tilde{q}}_{\mathrm{ih}}$ denote the estimate of the percentile profile ***Q*** for the *h*th bootstrap sample from the *i*th population (*i* = 1,⋯,*K* and *h* = 1,⋯,*B*). Further, let ${\tilde{q}}_{i}$ be the *B* × *p* matrix of bootstrap percentile estimates from the *i*th population. There is no restriction on the bootstrap sampling plan; we illustrate the method with standard *n* out of *n* sampling.
**Step 4.** Compute the *p × p* covariance matrix $V_{i}={\left(B-1\right)}^{-1}\left[{{\tilde{q}}^{\prime }}_{i}(I_{B}-B^{-1}11^{\prime }){\tilde{q}}_{i}\right]$ for *i* = 1,⋯, *K* where ***I_B_*** is the identity matrix of dimension *B* and **1** is the column vector of ones of length *B*. Then, arrange the variance-covariance matrices in an overall block-diagonal covariance matrix, ***V***, of dimension *Kp* × *Kp* where the off-diagonal matrices are **0**:
  $$V=\left[\begin{array}{ccc} V_{1} & 0 & 0\\ 0 & \ddots & 0\\ 0 & 0 & V_{K} \end{array}\right]$$
**Step 5.** Consider the *c* × *Kp* contrast matrix ***A*** where 1 ≤ *c* ≤ (*Kp* − 1). To test the null hypothesis *H*_0_: ***Aq*** = **0**, calculate the Wald test statistic
  (1) $$W=q^{\prime }A^{\prime }{\left(\mathrm{AVA}\prime \right)}^{-1}Aq\overset{D}{\to }\chi_{c}^{2}$$
**Step 6.** The 100 (1−*α*) % confidence interval for any linear function of a set of percentiles, say, ***aq***, where ***a*** is a 1 × *Kp* vector of constants, is calculated by
  (2) $$aq\pm z_{1-\alpha /2}{\left(\mathrm{aVa}\prime \right)}^{1/2}$$
  where *z*_1−_*_a/_*_2_ is the 1 − *a*/2 critical value of the standard normal distribution. In general, ***A*** is chosen to jointly test the hypotheses of interest and each ***a***, which could be an individual row of ***A***, is taken separately to construct confidence intervals on the individual population percentiles corresponding to the linear combinations created by ***A***. The level of α in Equation (2) may be adjusted for multiple tests using a method such as Bonferroni adjustment. The following numerical example illustrates some potential choices of ***a*** for generating confidence intervals of interest.

### 2.3. A Numerical Example for Two Populations

For illustrative purposes, consider the following example with simulated data with hypothetical values of ***q*** and ***V***. We are given two independent samples (*K* = 2) and wish to test the equality of three percentiles (*p* = 3) between them, which we will refer to as the percentile profile ***Q*** = (*Q*_1_, *Q*_2_, *Q*_3_). Suppose that we are interested in the 25th, 50th, and 75th percentiles; *i.e*., *u* = (0.25, 0.5, 0.75). Let ***q***_1_ = (5.04, 8.38, 11.21) and ***q***_2_ = (4.00, 6.28, 9.95) be the estimates of the first and second populations’ percentile profiles, respectively. Arranging the sample estimates in a column vector ***q***, we find

$$q^{\prime }=\left[5.04\;8.38\;11.21\;4.00\;6.28\;9.95\right]$$

Next we obtain *B* bootstrap sample estimates of ***Q*** for each population and calculate the variance-covariance matrix of the bootstrap sample estimates. Then we calculate ***V***, which is a *Kp* × *Kp* block-diagonal matrix with zeros (imposed by the assumption of independence) on the off diagonal blocks. In our example simulated data, we calculated ***V*** equal to

$$V=\left[\begin{array}{cccccc} 0.455 & 0.279 & 0.168 & 0 & 0 & 0\\ 0.279 & 0.519 & 0.264 & 0 & 0 & 0\\ 0.168 & 0.264 & 0.450 & 0 & 0 & 0\\ 0 & 0 & 0 & 0.264 & 0.183 & 0.185\\ 0 & 0 & 0 & 0.183 & 0.377 & 0.371\\ 0 & 0 & 0 & 0.185 & 0.371 & 1.162 \end{array}\right]$$

If we are interested in testing the equality of percentile profiles (***H***_0_: *q*_1_*_j_* = *q*_2_*_j_* for *j* = 1,⋯, *p*), we choose ***A*** = [***I****_p_*| −***I****_p_*]. With point estimates of the percentile profiles, ***q***, and estimated covariance matrix ***V***, we have everything necessary to jointly test the hypothesis *H*_0_: ***Q***_1_ = ***Q***_2_ as in Equation (1) and construct confidence intervals for ***Q***_1_ − ***Q***_2_ as in Equation (2). The Wald statistic is *W* = 4.97 with 3 degrees of freedom so we conclude the differences between the percentile profiles are not statistically significant. To construct confidence intervals on the differences of the percentiles between the groups, we follow Equation (2). Let ***a****_i_* be the necessary constant vector to construct the confidence interval for the difference between the *i*th percentiles of the two populations. We have ***a***_1_ = (1 0 0 −1 0 0), ***a***_2_ = (0 1 0 0 −1 0), and ***a***_3_ = (0 0 1 0 0 −1); *i.e*., the rows of ***A***. Using a Bonferroni adjustment on each estimate, the 95% confidence intervals are [−0.99, 3.07] for *Q*_11_ – *Q*_21_, [−0.17, 4.37] for *Q*_12_ – *Q*_22_, and [−1.78, 4.30] for *Q*_13_ – *Q*_23_.

Testing the equality of percentiles *q*_1_*_j_* = *q*_2_*_j_* for *j* = 1,⋯, *p* is just one of many possible contrasts we could construct. For example, we could test the equality of the inter quartile range (IQR), the difference of the 75th and 25th percentiles, between the two populations. To test this, we simply specify a different contrast matrix ***A***, which would be a 1 × *Kp* vector, where ***A*** = (−1 0 1 1 0 −1) ${\hat{R}}_{1}$ and ${\hat{R}}_{2}$ denote the estimate of the first and second population’s IQR and the difference as ${\hat{R}}_{1}-{\hat{R}}_{2}$. The variance estimate of ${\hat{R}}_{1}-{\hat{R}}_{2}$ is equal to

$$\begin{array}{l} \mathrm{Var}({\hat{R}}_{1}-{\hat{R}}_{2})=\mathrm{Var}({\hat{R}}_{1})+\mathrm{Var}({\hat{R}}_{2})-2\mathrm{Cov}({\hat{R}}_{1},{\hat{R}}_{2})\\ \;=\mathrm{Var}(q_{13}-q_{11})+\mathrm{Var}(q_{23}-q_{21})\\ \;=\mathrm{Var}(q_{13})+\mathrm{Var}(q_{11})-2\mathrm{Cov}(q_{13},q_{11})\\ \;+\mathrm{Var}(q_{23})+\mathrm{Var}(q_{21})-2\mathrm{Cov}(q_{23},q_{21}) \end{array}$$

This calculation is completed via matrix computations as in Equations (1) and (2). For the example above, ${\hat{R}}_{1}-{\hat{R}}_{2}=0.22$ and $\mathrm{Var}\left({\hat{R}}_{1}-{\hat{R}}_{2}\right)=1.625$. The calculation using Equation (1) yields a *W* of 0.03 so we would conclude that the IQR of the populations are not significantly different.

To test the equality of the IQR for *K* populations we generalize the above results. Let ***g*** be the 1 × *p* vector containing the contrast of interest. For testing the equality of the IQR with ***Q*** = (25, 50, 75), ***g*** = (−1 0 1). For 2 populations, only one comparison is necessary and ***A*** is of dimension 1 × *Kp*. However, for *K* > 1, we require *K* – 1 comparisons, with ***A*** in the form of

$$A=[\begin{array}{cccccc} g & -g & 0 & 0 & \cdots & 0\\ 0 & g & -g & 0 & \cdots & 0\\ 0 & 0 & g & -g & \cdots & 0\\ \vdots & \vdots & \vdots & \vdots & \ddots & \vdots \\ 0 & 0 & 0 & g & -g & 0\\ 0 & 0 & 0 & 0 & g & -g \end{array}]$$

where **0** is the 1 × *p* row vector of zeros. In this case, ***A*** is a (*K* – 1) × *Kp* matrix where the non-zero 1 × *p* vectors are ***A****_l,l_* = ***g*** and ***A****_l,l_*_+1_ = −***g*** for *l* = 1,⋯, *K* −1.

## 3. Simulation Studies

### 3.1. Accuracy of Bootstrap Variance Estimates

The convergence of bootstrap variance estimators of order statistics to the true variance has been demonstrated by Ghosh *et al*. [10] and Babu [11], with the exact rate of convergence and coverage error of confidence intervals shown by Hall and Martin in [5] and [6]. Empirical studies in the present paper demonstrate this behavior for the normal and gamma distributions. Two million replicate samples were generated and the (⌊*nu*⌋ + 1)th order statistics corresponding to the values of *u* listed in Table 1 (for comparing N(0,1) distributions) and Table 2 (for comparing gamma(shape = 2, scale = 1)) were found as the empirical estimate of the variance/covariance of the particular order statistics, denoted as $\bar{v}$. The normal distribution and gamma distributions were chosen to investigate differences in the bootstrap covariance estimation between symmetric and highly-skewed distributions. The particular parameters chosen for the gamma distribution yields relatively large values of skewness, which is desired. These empirical estimates were compared to bootstrap variance/covariance estimates (denoted as $\hat{v}$) of the same order statistics following steps 1 – 4 from Section 2 using *B* = 1000. All simulations were programmed and run in R 3.1.2.

In general, the relative error of the bootstrap variance/covariance estimates decreases as sample size increases. In some instances (in small sample sizes, such as 20) the variance increases as sample size increases. This occurs in the extreme percentiles in both the normal and gamma samples. This likely occurs because the order statistics used for *Q*(0.05) and *Q*(0.95) are close to 1 and *n*. In bootstrap resampling, this causes artificially low estimates of variance when sample sizes are small due to the limited number of unique values that the particular bootstrap order statistic may take. In these situations (*Q*(0.05) in normal samples, *Q*(0.05) and *Q*(0.95) in gamma samples) the expected behavior resumes at around *n* = 50. Also, it is important to note that all estimates of the variance of a single order statistic (excluding the 95th percentile estimate of gamma with *n* = 20) have positive bias while all estimates of covariance of two order statistics (excluding those that are arbitrarily small) have negative bias. It can be seen from Table 1 and Table 2 that the covariance estimates are considerably closer to being accurate than the variance estimates in terms of relative error. It would seem that because of the inflated bootstrap variance estimates that this would cause issues with the Wald test. In the next section further studies are shown to investigate the effects of the error of bootstrap variance and covariance estimates.

### 3.2. Performance of the Wald Test

Simulation studies were performed in which the Wald statistic was calculated for several percentile profiles that ranged from one up to nine equally-spaced percentiles. Other profiles were tested that included unevenly spaced percentiles with extreme percentiles in the tails of the distribution, referred to as ***Q***_1_ = (0.05, 0.95), ***Q***_2_ = (0.05, 0.25, 0.5, 0.75, 0.95), and ***Q***_3_ = (0.05, 0.1, 0.25, 0.5, 0.75, 0.9, 0.95), which specify the values of *u* in *q*(*u*). For example, testing one equally-spaced percentile would equate to the 50th percentile, or *u* = 0.5. Testing four percentiles would equate to testing the 20th, 40th, 60th, and 80th percentiles, or *u* = 0.2, 0.4, 0.6, 0.8. These percentile profiles, particularly ones with more than one percentile, are chosen to compare the populations across the entire sample space. Although the Wald statistic will vary depending on the profile chosen for a given sample, selecting a profile with similar values of *u* will cause only slight changes in the results of the test (more details later).

Again, the standard normal and gamma (2,1) distributions were used for empirical Type I error estimates to examine differences between symmetric (normal) and highly-skewed (gamma) distributions. As can be seen from the empirical type I error in Table 3 and Table 4, the shape of the distribution has little effect on the asymptotic behavior of the test as empirical Type I error is roughly the same under both scenarios. Also, the error of the bootstrap estimate of percentile variance appears to be substantially moderated when used in the Wald test. For example, the normalized error of the bootstrap variance estimate of the 5th percentile (the sixth order statistic for *n* = 100) in *N*(0,1) is 0.226 (from Table 1). It is assumed that the 95th percentile is roughly the same due to symmetry of the distribution though not exact due to the particular order statistics used. However, the empirical type I error of the Wald test comparing the 5th and 95th percentiles between the populations is 0.0483, which is unexpected considering the magnitude of the error of the bootstrap variance estimates. In another example, the normalized error of the bootstrap variance estimates of ***Q***_1_ = (0.05, 0.95) for *n* =100 from gamma (shape = 2, scale = 1) are 0.185 and 0.310, respectively (from Table 2). However, the Wald test yields an empirical type I error of just 0.0475.

The results in Table 1 through Table 4 indicate that the Wald test is robust to large error of the bootstrap variance estimates of the percentiles, at least in the sense that type I error is less than the target value, 0.05 in this case. Also, profiles with more percentiles require greater sample size before convergence to the theoretical alpha level. For the parameters chosen in Table 3 and Table 4, the empirical type I error is adequate for samples as few as 20 in most cases, and for profiles with extreme percentiles (*i.e*. 5th and 95th) the type I error is adequate between sample size 40 and 60. As a general rule of thumb, sample sizes of at least 50 will give reliable results for profiles of any size and any percentile.

As mentioned previously, the primary purpose of the test is to compare the distributions of random variables among populations along the variable’s entire domain but have the added precision of testing the equality of specific parameters, *i.e*., the percentiles. With certain percentile profiles, the test has similarities of both: (1) tests that are designed to detect any difference between distributions, such as the KS test, and (2) tests on specific population parameters, such as the t-test for means. For comparative purposes, there is no procedure to the authors’ knowledge that explicitly tests a set of percentiles among populations. Rank or location tests as well as global tests of equality are not appropriate comparisons; nevertheless, we used the KS test as a benchmark of the power under various distributions.

For each set of parameters for the distributions listed in Table 5 and Table 6, the sample size necessary for the KS test to achieve a power of 0.8 was found (based on 100,000 replicate samples). With the same sample size for the particular distributions, the proposed test was performed for various percentile profiles and the empirical power calculated based on 10,000 replicate samples. Results of the simulations that test equally-space percentile profiles are listed in Table 5 and unequally-spaced profiles are in Table 6. The test is competitive with the KS in most of the scenarios tested. With some distributions and percentile profiles, the test has greater power than the KS test. For equally-spaced percentiles, the profiles with 1, 2, 3, and 4 percentiles perform similarly, with the 2 and 3 percentile profiles having slightly greater average power across all scenarios. For unequally-spaced percentile profiles, profile ***Q***_2_ performed the best while the profile containing only the upper and lower extremes, ***Q***_1_, performed the worst. These simulation results outline a general strategy for using this procedure.

In general, there is not likely interest in a specific value of *u*, but there may be such interest in arbitrary values in the small neighborhood around *u*. For instance, in a test involving the median, we may not be really interested in the actual value at which half the observations are above and half below, but rather some measure of the general “center” of the distribution by which we could compare the two populations. Similarly, we could choose to test the equality of 25th or 20th percentile, not necessarily because this is a specific parameter of interest, but merely some measure of the lower half of the distribution. If we were to construct confidence intervals at each value of *q*(*u*), we could examine the extent of the differences at these exact locations, but likely interpret them as just a sample of the relationship between the distributions in the segment near *q*(*u*). Essentially, we are systematically sampling along the domain of the populations to detect differences between the distributions. Therefore, if there is not a specific profile of interest to be tested, the percentile profile should be selected to adequately cover the entire domain, particularly in the tails of the distributions where discrepancies frequently exist.

## 4. Illustrative Example: C-Reactive Protein and Diabetes

We demonstrate the use of the procedure with an illustrative example involving C-reactive protein (CRP) levels and diabetes using data from the 2009–2010 National Health and Nutrition Examination Surveys (NHANES). CRP is found in blood plasma and serves as a biomarker for inflammation. Physicians find it informative to monitor patients who are ill because CRP levels tend to rise with injury or disease progression and decline with healing or disease regression. Suppose we wished to test for differences in the distribution of CRP between groups based on diabetes status determined by plasma fasting glucose (PFG). Individuals with PFG (mg/dL) of 99 or below are classified as “normal”, between 100 and 125 are classified as “prediabetes”, and 126 or above as “diabetes”, as designated by the American Diabetes Association (http://www.diabetes.org/diabetes-basics/diagnosis/). We used the procedure to test that the 5th, 10th, 25th, 50th, 75th, 90th, and 95th percentiles were equal between (1) normal males and males with diabetes and (2) normal females and females with diabetes (Only data on adults between the ages of 18 and 79 were analyzed).

For males, there were 533 within the normal group and 160 in the diabetes group. The difference in percentile profiles between normal males and diabetes males is highly significant with a Wald test statistic of 42.9 with seven degrees of freedom (*p* < 0.0001). For females, there were 867 within the normal group and 123 in the diabetes group with a Wald statistic of 14.0 (*p* = 0.0517), indicating there is likely a difference between the groups’ percentile profiles. It is important to remember that alternate profiles will result in different values of Wald statistics from the differences in the sample order statistics as well as the distribution of the order statistics within the sample. Due to this uncertainty of estimates, a more informative analysis likely involves confidence intervals as seen in Table 7.

Let ***d*** represent the vector of differences between population percentile estimates, equal to ***q′A′*** where ***q*** is the combined vector of population percentile estimates and ***A*** = [***I****_p_*| − ***I****_p_*]. Let *d_i_* = *q_iD_* − *q_iN_* be the estimated difference between the *i*th percentile of the diabetes and normal groups. Recall from Equation (2) that the confidence interval for the difference is ***d_i_*** ± *z*_1–_*_α_*_/2_ (*a_i_Va′_i_*)^1/2^ where ***a****_i_* is the *i*th row of ***A***. In this case, with four comparisons, it would be appropriate to adjust *z*_1−_*_α_*_/2_ for each *d_i_* to ensure the family−wise error rate does not exceed 0.05; thus a Bonferroni correction was made. Estimates and confidence intervals are presented Table 7. By examining the differences and calculating confidence intervals at specified percentiles that span the entire domain, better insight into the differences between populations is possible. For both males and females, it is not a simple shift in location of CRP between normal and diabetes groups; there appears to be increasing differences as the percentile increases.

## 5. Conclusions

The Wald test provides a natural framework for testing equality of linear combinations of percentiles among multiple groups. Although estimating percentiles and obtaining estimates of variance via bootstrap samples has been previously investigated, the use of the Wald test for hypothesis testing has not been proposed. As shown with simulation studies, the procedure has excellent asymptotic properties and is readily implemented in practical applications as illustrated in this paper. The selection of a percentile profile, coupled with the ability to construct various contrasts, allows us to compare populations across the entire domain in detail and detect important differences at multiple locations.

The test may have limited use in some circumstances as the test relies on large-sample assumptions. Some profile/distribution combinations may yield inaccurate bootstrap estimates of percentile variance for small sample sizes. As shown in simulation studies, variance estimates of extreme percentiles (order statistics) near 1 and *n* are inaccurate in small sample sizes. However, these errors seem to have limited effect on the performance of the Wald test. With reasonable sample sizes, it is an excellent nonparametric procedure for population comparison and testing and is competitive with standard nonparametric tests for testing equality of distributions, specifically the two sample Kolmogorov-Smirnov test.

Another important aspect of the procedure is the choice of percentiles to be tested. If the exact percentiles are not important, a profile (with equally- or unequally-spaced percentiles) along the entire domain of the distribution is effective as an overall nonparametric test of global inequality, but can also be used to test the equality of linear combinations of the percentile profiles to obtain specific estimates at these locations. More research can be done to determine the optimal allocation of percentiles within a profile, perhaps based on features of the data. Further work can investigate the sample size limitations and distribution effect upon the bootstrap estimates of variance how this in turn affects the Wald test.

## Figures and Tables

**Table 1:** Error relative to empirical expected value of bootstrap variance-covariance estimates from *N*(0,1).

*n*	$(\hat{v}-\bar{v})/\bar{v}$								
	0.05	0.25	0.5	(0.05, 0.25)	(0.05, 0.5)	(0.05, 0.75)	(0.05, 0.95)	(0.25, 0.5)	(0.25, 0.75)
20	0.172	0.217	0.210	−0.110	−0.110	−0.109	−0.427	−0.032	−0.053
40	0.310	0.168	0.143	−0.044	−0.047	−0.035	−0.124	−0.026	−0.018
60	0.281	0.137	0.127	−0.031	−0.017	−0.020	−0.038	−0.019	−0.019
80	0.242	0.130	0.108	−0.023	−0.022	−0.027	−0.016	−0.015	−0.011
100	0.226	0.108	0.104	−0.023	−0.009	−0.007	−0.026	−0.014	−0.004
150	0.193	0.098	0.081	−0.016	−0.011	−0.001	−0.001	−0.010	0.000
200	0.157	0.085	0.079	−0.015	−0.008	−0.013	−0.016	−0.004	−0.005
250	0.151	0.081	0.078	−0.001	0.005	0.008	−0.002	0.002	0.004

**Table 2:** Error relative to empirical expected value of bootstrap variance-covariance estimates from gamma (2,1).

*n*	$(\hat{v}-\bar{v})/\bar{v}$								
	0.05	0.25	0.5	0.95	(0.05, 0.5)	(0.05, 0.95)	(0.25, 0.5)	(0.25, 0.75)	(0.25, 0.95)
20	0.210	0.231	0.243	−0.583	−0.079	−0.456	−0.013	−0.026	−0.441
40	0.227	0.170	0.171	0.242	−0.036	−0.086	−0.015	−0.012	−0.081
60	0.202	0.144	0.144	0.476	−0.030	−0.040	−0.012	−0.021	−0.020
80	0.187	0.123	0.125	0.391	−0.025	−0.014	−0.009	−0.010	−0.012
100	0.185	0.119	0.098	0.310	−0.016	−0.028	−0.013	0.000	−0.005
150	0.147	0.100	0.097	0.229	−0.018	−0.012	−0.004	−0.002	−0.010
200	0.143	0.085	0.081	0.208	−0.010	−0.020	−0.002	−0.001	−0.004
250	0.137	0.078	0.067	0.185	−0.006	0.038	−0.006	−0.001	0.007

**Table 3:** Empirical Type I error (10,000 replicates) of Wald Test from *N*(0,1).

*n*	Equally-spaced Percentiles						Unequally-spaced Percentiles		

	1	2	3	4	7	9	*Q*_1_	*Q*_2_	*Q*_3_
20	0.0484	0.0453	0.0332	0.0286	0.0182	0.0142	0.1919	0.1175	0.0786
40	0.0488	0.0455	0.0378	0.0324	0.0194	0.015	0.0652	0.0471	0.0334
60	0.0445	0.0432	0.0402	0.0349	0.0236	0.017	0.0456	0.0425	0.0313
80	0.0475	0.0461	0.0473	0.0403	0.0284	0.0204	0.0448	0.0407	0.031
100	0.0486	0.0499	0.0464	0.0439	0.029	0.0216	0.0483	0.0437	0.0325
150	0.0512	0.0477	0.0479	0.0461	0.0331	0.0261	0.048	0.0454	0.0394
200	0.0523	0.0513	0.049	0.0452	0.0371	0.0314	0.0475	0.0453	0.0338
250	0.0499	0.0492	0.0497	0.0447	0.0377	0.0322	0.0525	0.051	0.0415

**Table 4:** Empirical Type I error (10,000 replicates) of Wald Test with from gamma (shape = 2, scale = 1).

*n*	Equally-spaced Percentiles						Unequally-spaced Percentiles		

	1	2	3	4	7	9	*Q*_1_	*Q*_2_	*Q*_3_
20	0.0441	0.0405	0.034	0.0305	0.0187	0.0181	0.2111	0.1292	0.0844
40	0.0495	0.0457	0.0387	0.0321	0.0203	0.0147	0.0718	0.0512	0.0343
60	0.0473	0.0393	0.0381	0.0348	0.0233	0.0184	0.0483	0.0408	0.0288
80	0.051	0.0458	0.042	0.0358	0.0265	0.0173	0.0441	0.0401	0.0307
100	0.0494	0.0477	0.0435	0.0371	0.0279	0.02	0.0475	0.0388	0.0293
150	0.0482	0.0496	0.0443	0.044	0.0307	0.0248	0.0483	0.0435	0.0337
200	0.0507	0.0486	0.0453	0.0445	0.0352	0.0283	0.0501	0.048	0.0382
250	0.0517	0.0506	0.0471	0.0462	0.0377	0.0314	0.0508	0.0487	0.0382

**Table 5:** Simulation results compared to Kolmogorov-Smirnov test at 80% power with equally-spaced percentiles.

			Equally-spaced Percentiles					

Distributions	*n*	Power	1	2	3	4	7	9
Gam (2, 1) & Gam (2.4, 1)	241	0.7999	0.7112	0.7248	0.7159	0.6959	0.6466	0.6203
Gam (2, 1) & Gam (2.2, 1.2)	138	0.8024	0.718	0.7416	0.7293	0.712	0.6675	0.6335
Gam (2, 1) & Gam (2.3, 1.3)	70	0.7997	0.7425	0.7717	0.7665	0.7547	0.7069	0.6629
Gam (2, 1) & Gam (2.4, 1.4)	41	0.7936	0.7213	0.7568	0.7546	0.7357	0.6743	0.6148
Gam (4, 1) & Gam (4.2, 1.2)	104	0.8033	0.7249	0.7428	0.7351	0.7231	0.6701	0.6339
Gam (4, 1) & Gam (4.3, 1.3)	50	0.7945	0.7117	0.7442	0.7326	0.7178	0.653	0.6099
Gam (2, 1) & N (2.2, 1)	101	0.7971	0.7016	0.7763	0.8026	0.8091	0.7999	0.7671
Gam (2, 1) & N (2.3, 1.2)	74	0.8051	0.6943	0.7754	0.7897	0.796	0.7588	0.7264
Gam (2, 1) & N (2.3, 1.2)	102	0.7973	0.7697	0.7512	0.738	0.7124	0.6834	0.6454
Average Power		0.7992	0.7217	0.7539	0.7516	0.7396	0.6956	0.6571

**Table 6:** Simulation results compared to Kolmogorov-Smirnov test at 80% power with unequally-spaced percentiles.

Distributions	*n*	Power	Unequal Percentiles		
			*Q*_1_	*Q*_2_	*Q*_3_
Gam (2, 1) & Gam (2.4, 1)	241	0.7999	0.4766	0.6911	0.6422
Gam (2, 1) & Gam (2.2, 1.2)	138	0.8024	0.464	0.7015	0.6682
Gam (2, 1) & Gam (2.3, 1.3)	70	0.7997	0.4929	0.745	0.6916
Gam (2, 1) & Gam (2.4, 1.4)	41	0.7936	0.5265	0.7404	0.6814
Gam (4, 1) & Gam (4.2, 1.2)	104	0.8033	0.4776	0.7133	0.6653
Gam (4, 1) & Gam (4.3, 1.3)	50	0.7945	0.4712	0.7174	0.6605
Gam (2, 1) & N (2.2, 1)	101	0.7971	0.3522	0.862	0.8335
Gam (2, 1) & N (2.3, 1.2)	74	0.8051	0.2832	0.8141	0.7796
Gam (2, 1) & N (2.3, 1.2)	102	0.7973	0.0809	0.774	0.7064
Average Power		0.7992	0.4028	0.7510	0.7032

**Table 7:** Estimates of C-reactive protein differences in percentiles between diabetes and normal groups for males and females.

Percentile	Males			Females		

	*d*	Std. Error	95% Confidence Interval	*d*	Std. Error	95% Confidence Interval
5th	0.03	0.0098	0.0035, 0.0565	0.02	0.0102	−0.0075, 0.0475
10th	0.03	0.0065	0.0124, 0.0476	0.03	0.0110	0.0004, 0.0596
25th	0.05	0.0132	0.0145, 0.0855	0.04	0.0213	−0.0175, 0.0975
50th	0.13	0.0310	0.0466, 0.2134	0.11	0.0438	−0.0080, 0.2280
75th	0.27	0.0803	0.0535, 0.4865	0.26	0.1066	−0.0274, 0.5474
90th	0.46	0.1311	0.1067, 0.8133	0.36	0.1732	−0.1069, 0.8269
95th	0.07	0.2713	−0.6613, 0.8013	0.29	0.5538	−1.2028, 1.7828

## References

[1] Johnson WD, Beyl RA, Burton JH, Johnson CM, Romer JE, Zhang L (2015). Use of Pearson’s Chi-Square for Testing Equality of Percentile Profiles across Multiple Populations. Open Journal of Statistics.

[2] Maritz JS, Jarrett RG (1978). A Note on Estimating the Variance of the Sample Median. Journal of the American Statistical Association.

[3] Efron B (1979). Bootstrap Methods: Another Look at the Jackknife. The Annals of Statistics.

[4] Bickel PJ, Freedman DA (1981). Some Asymptotic Theory for the Bootstrap. The Annals of Statistics.

[5] Hall P, Martin MA (1988). Exact Convergence Rate of Bootstrap Quantile Variance Estimator. Probability Theory and Related Fields.

[6] Hall P, Martin MA (1991). On the Error Incurred Using the Bootstrap Variance Estimate When Constructing Confidence Intervals for Quantiles. Journal of Multivariate Analysis.

[7] Cheung KY, Lee SM (2005). Variance Estimation for Sample Quantiles Using the *m* out of *n* Bootstrap. Annals of the Institute of Statistical Mathematics.

[8] Wilcox RR (1995). Comparing Two Independent Groups via Multiple Quantiles. Journal of the Royal Statistical Society, Series D.

[9] Hutson AD, Ernst MD (2000). The Exact Bootstrap Mean and Variance of an *L*-Estimator. Journal of the Royal Statistical Society, Series B.

[10] Ghosh M, Parr WC, Singh K, Babu GJ (1985). A Note on Bootstrapping the Sample Median. The Annals of Statistics.

[11] Babu GJ (1986). A Note on Bootstrapping the Variance of Sample Quantile. Annals of the Institute of Statistical Mathematics.

